# Phenotype Similarity Regression for Identifying the Genetic Determinants of Rare Diseases

**DOI:** 10.1016/j.ajhg.2016.01.008

**Published:** 2016-02-25

**Authors:** Daniel Greene, NIHR BioResource, Sylvia Richardson, Ernest Turro

**Affiliations:** 1 Department of Haematology, University of Cambridge, NHS Blood and Transplant, Cambridge Biomedical Campus, Cambridge CB2 0PT, UK; 2 Medical Research Council Biostatistics Unit, Cambridge Biomedical Campus, Cambridge CB2 0SR, UK

## Abstract

Rare genetic disorders, which can now be studied systematically with affordable genome sequencing, are often caused by high-penetrance rare variants. Such disorders are often heterogeneous and characterized by abnormalities spanning multiple organ systems ascertained with variable clinical precision. Existing methods for identifying genes with variants responsible for rare diseases summarize phenotypes with unstructured binary or quantitative variables. The Human Phenotype Ontology (HPO) allows composite phenotypes to be represented systematically but association methods accounting for the ontological relationship between HPO terms do not exist. We present a Bayesian method to model the association between an HPO-coded patient phenotype and genotype. Our method estimates the probability of an association together with an HPO-coded phenotype characteristic of the disease. We thus formalize a clinical approach to phenotyping that is lacking in standard regression techniques for rare disease research. We demonstrate the power of our method by uncovering a number of true associations in a large collection of genome-sequenced and HPO-coded cases with rare diseases.

## Introduction

There is widespread interest in the study of rare diseases as a way of understanding the genetic architecture of biological processes. Consequently, tens of thousands of subjects are being phenotyped extensively and enrolled to genome-sequencing studies worldwide. To discover the cause of disease, these subjects would ideally be grouped a priori into clusters with a shared (though unknown) genetic etiology, but this is often hindered by extensive phenotypic and genetic heterogeneity (see Web Resources and examples^1^, ^2^, ^3^, ^4^, ^5^, ^6^, ^7^, ^8^, ^9^). Rare variant association tests, even those accounting for some degree of genetic heterogeneity, typically summarize the clinical manifestations of a disease with a single variable,^10^ which can limit power when multiple phenotypic traits contain complementary information about the same causal genotype. Methods for modeling pleiotropy have proven successful in the context of genome-wide association studies^11^, ^12^ but they are ill suited for rare disease studies in which the phenotype data are typically of mixed type and collected with variable detail and completeness.

The Human Phenotype Ontology (HPO)^13^ addresses the need for a standardized vocabulary for rare disease phenotypes and is being used to code patients in several large international projects^14^, ^15^, ^16^ (see also Web Resources). The HPO is a directed acyclic graph representing more than 10,000 phenotypic abnormalities in which the nodes (HPO terms) are connected to each other through “is-a” relations, represented as edges. The HPO was created with the support of experts in many areas of medicine to accommodate coding of phenotypic data derived from diverse sources, such as laboratory assays, images, graphs, and clinical interpretation. Methods exist that compare patient HPO data with HPO-coded profiles corresponding to known diseases for the purpose of differential diagnosis.^17^, ^18^ The HPO-coded profiles can be supplemented with functional gene-specific information to prioritize genes.^19^, ^20^ If genotype data are available, these and other methods^21^, ^22^ can be used to prioritize variants and potentially to suggest new causes of disease.^19^, ^20^, ^23^ However, the existing approaches do not share information between individually coded patients and as such are not statistical association methods.

Here, we present a regression-based method for discovering associations between arbitrarily diverse sets of HPO-coded phenotypes and genotypes at rare variant sites. To overcome the difficulty of modeling sparse and ontologically structured phenotype data, we treat the HPO-coded phenotypes of the subjects as the explanatory variables and their corresponding genotypes as the response. This is an example of “inverse regression” and is adequate in our setting because we are not interested in interpreting the regression coefficients per se but only in evaluating the probability of association. We define a subject’s “genotype” *y* as a binary label that can take on the values “rare” (1) or “common” (0) according to a pre-specified function of the genetic data. For example, we could define the label “rare genotype” to mean that there is at least one rare variant in a particular gene (dominant inheritance) or at least two rare variants in a particular gene (recessive inheritance).

Our method then seeks to compare two models for the data, indexed by *γ*. Under the baseline model (*γ* = 0), the probability of observing the rare genotype is the same for each case. Under the alternate model (*γ* = 1), the probability of observing the rare genotype depends on the “phenotypic similarity” *S* (to be defined later) of the case to a latent *characteristic* HPO phenotype *ϕ*.

We adopt a Bayesian inference framework, where the model selection indicator *γ* and characteristic phenotype *ϕ* are estimated through their posterior distributions. Of particular interest is the posterior mean of *γ*, which represents the probability that *γ* = 1, thus indicating the strength of evidence for an association.

A crucial element of our approach is the construction of an appropriate function for quantifying the semantic similarity of the characteristic phenotype *ϕ* to the phenotypes of the subjects. The choice of function is motivated by the need to optimally discriminate between subjects having clinical features that are pertinent to a disorder from those having overlapping or unrelated phenotypes due to a different disorder. To achieve this, we have chosen a function that accounts for the ontological structure of the HPO and induces a parsimonious characteristic phenotype: it selects the required terms to distinguish patient groups while avoiding overfitting and is robust to coding of patients with spurious or sporadic terms. Importantly, the function is flexible with respect to the phenotypic variability of disease and robust to the HPO coding practices of clinicians.

Our Bayesian approach provides a natural means of incorporating information from the scientific literature into our prior belief about the characteristic phenotype. In this work, we focus on gene-specific inference and up-weight the prior probability of characteristic phenotypes that are similar to clinical^23^ and murine phenotypes^24^ relevant to the gene.

We demonstrate the effectiveness of our method in identifying associations between genotype and phenotype through a simulation study, whereby phenotypes are simulated given genotypes in such a way as to emulate the effect of a hypothetical set of pathogenic variants. We go on to apply our inference procedure to a real dataset of more than 2,000 unrelated individuals enrolled to a variety of rare-disease sequencing studies under the auspices of the BRIDGE projects run by the NIHR BioResource – Rare Diseases (Web Resources). We show that our method, implemented in the SimReg software package, can identify genes with rare variants responsible for a diverse set of pathologies in a single application and can estimate recognized disease phenotypes.

## Material and Methods

### Model Specification

We use a logistic regression framework to specify the two models under comparison:

(Equation 1) $$\begin{array}{c} y_{i}∼\text{Bernoulli}\left(p_{i}\right),\\ \gamma =0:\;\text{log}\left(\frac{p_{i}}{1-p_{i}}\right)=\alpha ,\\ \gamma =1:\;\text{log}\left(\frac{p_{i}}{1-p_{i}}\right)=\alpha +\beta S\left(\phi ,x_{i}\right). \end{array}$$

**Figure 1:**
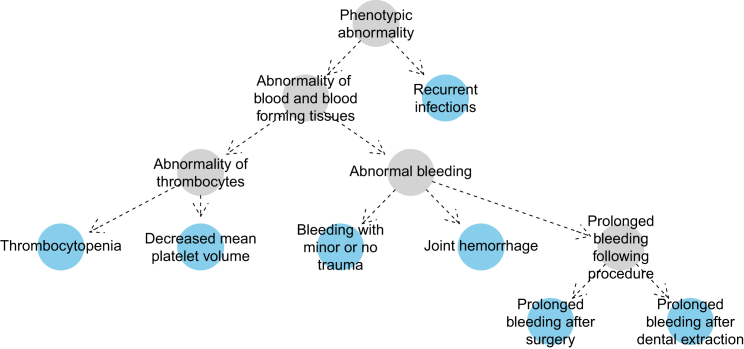
Example HPO Coding of a Subject with Wiskott-Aldrich Syndrome The nodes in blue imply the presence of the more general ancestral phenotypes depicted as gray nodes. No blue node has a directed path to any other, which means that the blue nodes comprise a *minimal* set of HPO terms. The graph has been simplified by removing nodes that link together only two other nodes.

Here, *y*_1_, ..., *y*_*N*_ are the genotypes of the *N* subjects in the collection, where *y*_*i*_ = 1 if subject *i* possesses the rare genotype and *y*_*i*_ = 0 if subject *i* possesses the common genotype. *x*_1_, ..., *x*_*N*_ are the corresponding phenotypes of the subjects, where *x*_*i*_ comprises the minimal set of HPO terms required to describe the phenotypic abnormalities of subject *i*. Loosely speaking, a set of terms is minimal if it describes a patient’s phenotype without redundancy (e.g., it does not include both “Abnormal bleeding” and “Joint hemorrhage”). More formally, a set of terms is said to be minimal if and only if it lacks elements implied by other terms in the set through directed edges in the HPO. The terms highlighted blue in Figure 1 comprise such a set, because there is no directed path between any pair of blue nodes.

The term *S*(*ϕ*, *x*_*i*_) denotes a chosen measure of phenotypic similarity between the characteristic phenotype and subject *i*’s phenotype. Note that our response and predictor are inverted compared to classical regression methods to avoid having to treat sparse and structured HPO data as the response. Under the baseline model, the intercept *α* is the global rate of rare genotypes. Under the alternate model, there is an additional parameter *β*, which is strictly positive and captures the effect of a unit increase in phenotypic similarity to the characteristic phenotype *ϕ* on the log odds of having the rare genotype. Thus, the probability that *γ* = 1 is greater in expectation when *S*(*ϕ*, *x*_*i*_) is larger if *y*_*i*_ = 1 than if *y*_i_ = 0.

### Similarity Measure

Our chosen similarity measure *S* is built with consideration for (1) quantification of the similarity of terms, (2) quantification of the similarity of a patient phenotype *x*_*i*_ to the characteristic phenotype *ϕ*, and (3) flexible transformation of the similarity between phenotypes.

Consistent with the ontological literature, we base our measure for the similarity of terms on the information content (IC) of each individual term,

IC(t)=−log(frequency(t)),

where the frequency of term *t* can be derived from its appearance in the case collection, including instances in which this is implied by the presence of more specific terms in the ontology.

We use Lin’s^25^ similarity function to compare two different terms:

$$s\left(t_{1},t_{2}\right)=\frac{2\times {\text{max}}_{t\in \text{anc}\left(t_{1}\right)\cap \text{anc}\left(t_{2}\right)}\text{IC}\left(t\right)}{\text{IC}\left(t_{1}\right)+\text{IC}\left(t_{2}\right)},$$

where anc(*t*) denotes the union of term *t* and its ancestral nodes in the HPO graph. For example, for the hypothetical subject shown in Figure 1, the expression ${\text{max}}_{t\in \text{anc}\left(t_{1}\right)\cap \text{anc}\left(t_{2}\right)}\text{IC}\left(t\right)$ if *t*_1_ were “Thrombocytopenia” and *t*_2_ were “Joint hemorrhage” would correspond to the IC of “Abnormality of blood and blood-forming tissues.” Because terms cannot have a higher IC than their descendants, the similarity *s* between two terms can range between zero and one. Next, we consider asymmetric measures of similarity between a case phenotype and *ϕ* inspired by the best-match-average (BMA) function,^17^ which computes the best match for each term and takes the mean:

$$S_{\phi }\left(\phi \to x_{i}\right)=\frac{1}{\left|\phi \right|}\sum_{t_{\phi }\in \phi }\underset{t_{x}\in x_{i}}{\text{max}}\;s\left(t_{\phi },t_{x}\right)1_{t_{\phi }\in \text{anc}\left(t_{x}\right)},$$

$$S_{x}\left(x_{i}\to \phi \right)=\frac{1}{\left|x_{i}\right|}\sum_{t_{x}\in x_{i}}\underset{t_{\phi }\in \phi }{\text{max}}\;s\left(t_{x},t_{\phi }\right)1_{t_{\phi }\in \text{anc}\left(t_{x}\right)}.$$

The standard BMA function does not include the indicator variable above, which evaluates to 1 only if the node in *ϕ* is among the ancestors of the node in *x*_*i*_. We prefer to include this restriction, which penalizes similarity to *ϕ* when it includes over-specific terms, in order to concentrate the posterior weight of *ϕ* preferentially on nodes that are present among the subjects.

The presence of a term in *ϕ* that is absent from *x*_*i*_ has the effect of lowering *S*_*ϕ*_, whereas the presence of a term in *x*_*i*_ that is absent from *ϕ* has the effect of lowering *S*_*x*_. Summation of two asymmetric similarities, as used in BMA, would allow reasonably high overall similarities to be obtained even when one of the two asymmetric similarities is close to zero. We prefer to multiply rather than add up the two similarity measures to obtain an expression for the overall similarity function used in Equation 1 because it ensures that the overall similarity can be high only when there is a high asymmetric similarity in both directions. However, because the values of *S*_*x*_ and *S*_*ϕ*_ are influenced by factors such as how frequent terms are in the reference database (which affects nodal IC) and the structure of the HPO graph, there is no guarantee that a linear function of their product optimally distinguishes subjects with objectively distinct clinical features. To ensure the model is robust to the choice of *S*, we allow modulation of the shapes of the similarity parameters, *S*_*ϕ*_ and *S*_*x*_, through transformations *f* and *g*, respectively. A reasonable choice for *f* and *g* is the beta cumulative distribution function (CDF), because it maps [0,1] to [0,1] monotonically and allows a wide variety of shapes:

$$f\left(z,\;a_{f},\;b_{f}\right)=I_{z}\left(a_{f},\;b_{f}\right),$$

$$g\left(z,\;a_{g},\;b_{g}\right)=I_{z}\left(a_{g},\;b_{g}\right),$$

where *I*_*z*_ is the regularized incomplete beta function (see Supplemental Note) and *a*_*f*_, *a*_*g*_, *b*_*f*_, and *b*_*g*_ are unknown parameters to be estimated.

Finally, the overall similarity function is given by

(Equation 2) $$S\left(\phi ,x_{i}\right)=f\left(S_{\phi }\left(\phi \to x_{i}\right),\;a_{f},\;b_{f}\right)\cdot g\left(S_{x}\left(x_{i}\to \phi \right),\;a_{g},\;b_{g}\right).$$

### Priors

We propose the following prior distributions for the model indicator and the regression parameters:

γ∼Bernoulli(π),

α∼Normal(mean=0,sd=5),

logβ∼Normal(mean=2,sd=1).

The value of *π* indicates how likely we believe a priori that there is a true association. All of the analyses in this paper assume *π* = 0.05. We place a vague prior on *α* around 0. Additionally, we include an offset on *α* by a constant ${\hat{h}}_{i}$ for each individual that can take into account batch effects and factors affecting the background rate of rare genotypes (not shown in Equation 1 for clarity of exposition, see Supplemental Note). The prior distribution on *β* is positive because the probability of *y*_*i*_ = 1 increases with *S*(*ϕ*,*x*_*i*_), given *γ* = 1. The prior variance of *β* allows for a wide range of effect sizes given the range of *S*. The priors on the beta CDF transformations are discussed in the Supplemental Note. In brief, the choice of prior for *f* favors parsimonious characteristic phenotypes and the prior for *g* allows for an indeterminate number of nodes appearing sporadically among patients.

By default, our prior distribution on the characteristic phenotype *ϕ* places a uniform prior probability on all minimal sets of terms of size less than or equal to *k*. We choose *k* = 3 on the grounds that three nodes should adequately distinguish between the primary features of most rare diseases. If *y* is set based on variants in a particular feature, such as a gene, then our prior can up-weight HPO phenotypes comprising terms annotated to that feature on the basis of reports in the scientific literature. Thus, the prior on *ϕ* is given by

(Equation 3) $$\mathbb{P}\left(\phi \right)=\{\begin{array}{cc} \frac{1}{\left|{\text{Φ}}^{\left(k\right)}\right|} & \text{No literature phenotype}\\ \frac{S\text{'}\left(M\to \phi \right)}{\sum_{\psi \in {\text{Φ}}^{\left(k\right)}}S\text{'}\left(M\to \psi \right)} & \text{Literature phenotype }M \end{array}$$

where ${\text{Φ}}^{\left(k\right)}$ denotes the set of all minimal sets of up to *k* HPO terms and *S*′ is an unstandardized similarity function (see Supplemental Note). In practice, the literature phenotype could be obtained from OMIM or from the Mouse Genome Informatics (MGI) database^24^ after mapping murine phenotypes coded using the Mammalian Phenotype Ontology^26^ to HPO terms through a cross-species phenotype ontology.^27^

### Inference

We perform model comparison using the Markov chain Monte Carlo (MCMC)-based method of Carlin and Chib.^28^ We sample the model selection parameter *γ* from its full conditional distribution while the remaining parameters are sampled using the Metropolis-Hastings algorithm or from a pseudoprior distribution, depending on the value of *γ* at each iteration.

It is not straightforward to sample from the space of minimal sets ${\text{Φ}}^{\left(k\right)}$ when *γ* = 1 because not all possible HPO term combinations comprise such a minimal set. To overcome this difficulty, we propose an unrestricted vector of *k* HPO terms $\tilde{\phi }$ and then derive the associated underlying phenotype *ϕ* by applying a mapping function *υ*. We therefore need to impose a prior distribution on the unrestricted space which is compatible with the desired prior for *ϕ* (Equation 3) on the restricted space. To be precise, the prior on $\tilde{\phi }$ is given by:

$$\mathbb{P}\left(\tilde{\phi }\right)=\frac{\mathbb{P}\left(\upsilon \left(\tilde{\phi }\right)\right)}{\left|\left\{\tilde{\phi }\text{'}\in {\text{H}}^{k}:\upsilon \left(\tilde{\phi }\text{'}\right)=\upsilon \left(\tilde{\phi }\right)\right\}\right|},$$

where H^*k*^ is the space of all vectors of *k* HPO terms and *υ* maps an arbitrary such vector of terms to its corresponding minimal set. The denominator accounts for the number of unrestricted vectors that map to the same minimal set. For further details on the method used to calculate ℙ(ϕ) and $\mathbb{P}\left(\tilde{\phi }\right)$, the MCMC algorithm, and the tuning of the pseudopriors, refer to the Supplemental Note.

## Results

### Simulation Study

We assessed the performance of SimReg by analyzing datasets generated under two scenarios, labeled by $\tilde{\gamma }$. Under $\tilde{\gamma }=1$, the HPO phenotypes *x*_1,...,*N*_ were simulated conditional on the genotypes *y*_1,...,*N*_ of *N* individuals whereas under $\tilde{\gamma }=0$ they were simulated independently of the genotypes. When $\tilde{\gamma }=1$, phenotypes for all subjects having *y*_*i*_ = 1 were formed by selecting terms from an arbitrarily chosen disease template (“Decreased mean platelet volume,” “Thrombocytopenia,” and “Autism”). Each term was selected with a pre-specified probability *r*, termed “expressivity,” and *m* further noise terms drawn at random from a set of approximately 1,000 HPO terms were appended, where *m* ∼ Poisson(*λ* = 5). The set of terms from which the noise terms were drawn was created by selecting 200 HPO terms at random, taking the union with the disease template terms, and then aggregating all the ancestral terms. Phenotypes for subjects having *y*_*i*_ = 0 were drawn at random using terms from the above set with *λ* = 8 and then mapped to minimal sets. When $\tilde{\gamma }=0$, all phenotypes were sampled from the noise term set with *λ* = 8. This ensures that on average individuals have approximately 8 terms, irrespective of *y*_*i*_ and $\tilde{\gamma }$. The simulation was performed with the set of disease template terms and set of noise terms fixed but with different numbers of individuals carrying the rare genotype $\left(\sum_{i}y_{i}\in \left\{2,4,6,8,10,20\right\}\;\text{out}\;\text{of}\;N=1,000\right)$ and varied levels of expressivity r∈{1/3,2/3,1}. The low expressivity set-ups capture situations in which a fraction of the individuals having a rare genotype can be considered to carry a neutral variant with respect to the disease in question because they have none of the template terms. For the same reason, they capture scenarios of incomplete penetrance of a subset of the underlying rare variants. Furthermore, a degree of genetic heterogeneity is built into our simulation setup, because there is a non-zero probability of a template phenotype term being randomly allocated to an individual with the common genotype.

**Figure 2:**
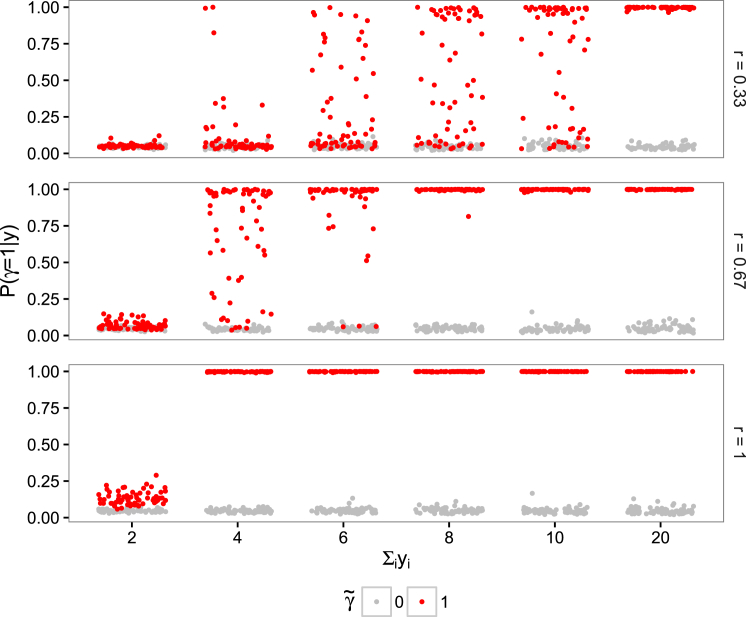
Results of Inference on Simulated Data Phenotype data were simulated using three levels of expressivity *r* of the disease terms. The plots within each panel correspond to different frequencies $\sum_{i}y_{i}$ of the rare genotype. In each plot, the red dots mark the estimated posterior mean of γ for 64 datasets simulated under $\tilde{\gamma }=1$ and the gray dots show an equivalent set of estimates for datasets simulated under $\tilde{\gamma }=0$ (i.e., whereby phenotypes for subjects having *y*_*i*_ = 1 are sampled from the same distribution as for subjects having *y*_*i*_ = 0). The position of points on the x axis within a plot is arbitrary.

The results of repeating the simulation 64 times for each value of $\tilde{\gamma }$ and combination of *r* and $\sum_{i}y_{i}$, depicted in Figure 2, show that power to detect a true association, as assessed by the posterior mean of γ, increases with the expressivity of the disease terms *r* and also with the frequency of the rare genotype in the study sample $\sum_{i}y_{i}$ (red dots). Under $\tilde{\gamma }=0$, the posterior mean of γ remains very close to zero in all circumstances (gray dots). Specifically, we find that 2, 6, and 20 cases out of 1,000 subjects are sufficient to obtain perfect or near-perfect discrimination between the two models when the expressivity is 1, 2/3, and 1/3, respectively. When the number of subjects with the rare genotype is equal to 6 and the expressivity is 2/3, which implies that any two individuals with the rare genotype have only a 0.17 chance of having exactly the same template terms, our method can achieve a positive predictive value of 1, even when the negative predictive value is as high as 0.95, by thresholding at ℙ(γ=1|y)≥0.25. Under this set-up, we expect 1.78 of the 6 individuals with the rare genotype to have none of the template terms at all, which indicates that the method has some resilience to the presence of *y*_*i*_ = 1 induced by neutral rather than pathogenic variants. In order to assess the specificity of the method more accurately, we have simulated 20,000 datasets under the scenario in which there is no association and $\sum_{i}y_{i}=6$ and found that only 7 datasets yield ℙ(γ=1|y)>0.25, which equates to a specificity of 99.97% for this chosen cut-off (Supplemental Note). We have also extended our simulation study to include a variable controlling genetic heterogeneity, whereby many individuals are drawn from the same template but only a subset have the rare genotype. Power is maintained even in challenging scenarios in which there is substantial genetic heterogeneity and moderate phenotypic expressivity (Supplemental Note). Overall, the results of our simulation study show that our method produces accurate results even in the presence of significant phenotypic or genetic heterogeneity and low expressivity of the rare genotype’s characteristic terms. Because these are typical hallmarks of many rare disease studies, our evaluation substantiates the utility of our approach.

### Results from Real Data

Our dataset comprises HPO phenotypes and corresponding variant call data for 2,045 unrelated individuals enrolled to a variety of rare-disease sequencing studies (Table 1). Detailed HPO data were available only for subjects enrolled to the Bleeding and Platelet Disorders (BPD) project.^14^ BPDs are a heterogeneous group of diseases, including polysymptomatic examples, making them an interesting use-case for the modeling we present. For the other projects, only high-level HPO terms were used (Table 1). A set of genes within which variants are known to be implicated in each class of disorders was provided by BRIDGE collaborators to assess the performance of the model (Supplemental Note).

We used variant call data from 686 sequenced exomes and 1,359 sequenced whole genomes. To account for biases that might alter the baseline rate of rare genotypes (e.g., sequencing platform), we use a plug-in offset in the regression Equation 1, estimated a priori (see Supplemental Note). Variants were retained only if they were predicted to alter protein sequence and were either absent from ExAC (Web Resources) or had an allele frequency therein below 1/1,000 or 1/10,000 when a recessive or dominant mode of inheritance, respectively, was assumed in the analysis. Rare variants were aggregated within genes to account for genetic heterogeneity and increase power. We defined the binary genotypes *y* based on three different aggregation approaches corresponding to the following hypothetical modes of inheritance: (1) dominant, i.e., presence of at least one rare allele; (2) recessive, i.e., presence of at least two rare alleles; or (3) high-impact dominant, i.e., presence of at least one rare allele predicted^29^ to introduce a splice site aberration, frameshift, start loss, or stop gain.

### *ACTN1* as Exemplar Gene

**Figure 3:**
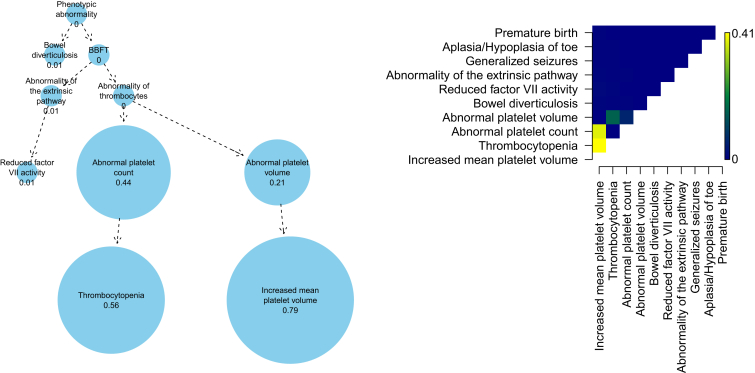
Results for *ACTN1* The panels show results obtained by applying the SimReg method to phenotype data for all subjects and genotype data for *ACTN1*. There were 43 individuals in our dataset coded with the rare genotype for this gene, of which 22 were coded with “Thrombocytopenia” and “Increased mean platelet volume.” The graph shows the estimated probabilities of inclusion of individual terms in *ϕ* (only the seven terms with the highest probabilities of inclusion and their ancestors are shown). The acronym “BBFT” refers to “Abnormality of blood and blood-forming tissues.” The heat map shows the estimated probabilities of pairs of terms co-occurring in *ϕ*, for pairs composed from the ten most frequently included individual terms.

We now describe the properties of SimReg’s output by focusing on a gene, *ACTN1* (MIM: 102575), that has recently been reported to harbor rare variants responsible for reduced platelet number and increased platelet size (macrothrombocytopenia).^30^ We note that data for *ACTN1* were used to inform and motivate our choice for the similarity measure given in Equation 2 (Supplemental Note). Once learnt on the *ACTN1* data, this choice has then been used universally for all genes. We observe strong evidence that the rare genotype for *ACTN1* is associated with similarity to a characteristic phenotype (ℙ(γ=1|y)=1), as expected. The estimated characteristic phenotype focuses primarily on phenotypes that include “Thrombocytopenia” and “Increased mean platelet volume” (Figure 3), which together correspond to macrothrombocytopenia. The slightly more general terms “Abnormal platelet count” and “Abnormal platelet volume” also have substantial marginal posterior weight whereas the rest of the nodes in the HPO have a marginal posterior probability of inclusion less than 0.02. As can be seen in a two-dimensional matrix of the marginal posterior on pairs of terms (Figure 3), there is a high degree of co-occurrence of the two primary terms representing the *ACTN1*-related phenotype, which implies that they are not alternatives but rather complements that together produce a good model fit.

### *DIAPH1* and *RASGRP2*

**Figure 4:**
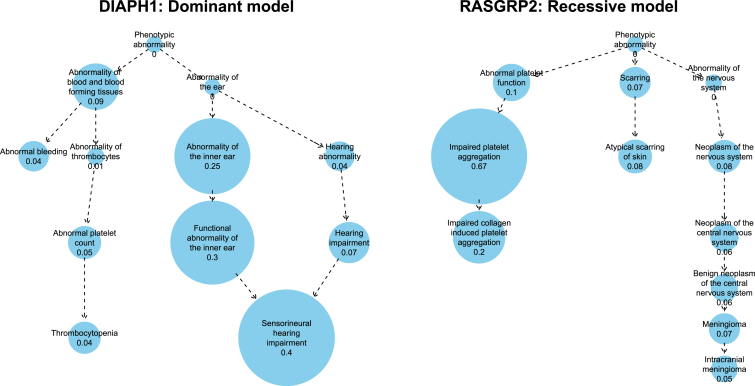
Results for *DIAPH1* and *RASGRP2* Estimated posterior probabilities of individual terms being included in the characteristic phenotype *ϕ* using phenotype data for all subjects and variant data for *DIAPH1*$\left(\sum_{i}y_{i}=2\right)$ encoded under a high-impact dominant model and *RASGRP2*$\left(\sum_{i}y_{i}=7\right)$ encoded under a recessive model. The ten terms with the highest marginal posterior probability are shown. The estimated posterior probability that *γ* = 1 is equal to 0.872 and 0.750 for *DIAPH1* and *RASGRP2*, respectively.

Under the high-impact dominant mode of inheritance described above, one of the genes with the highest estimated value of *γ* that also has a BPD-like inferred phenotype is *DIAPH1* (MIM: 602121) (*γ* = 0.87). We recently showed, through an application of our similarity regression approach, that the introduction of a premature stop codon present in two unrelated individuals in the BPD project truncates DIAPH1’s 3′ auto-inhibitory domain and causes macrothrombocytopenia, hearing loss, and mild bleeding.^31^ As shown in Figure 4 (left), the salient terms in *ϕ* relate to hearing impairment and abnormality of blood and blood-forming tissues, with the latter driven mainly by thrombocytopenia and bleeding. The high posterior estimate of *γ* was obtained in part because a sensorineural hearing loss phenotype had previously been reported in the literature,^32^ which up-weighted hearing abnormality terms in the prior for *ϕ* (Table 2). However, even without using an informative prior on *ϕ*, a high posterior probability of an association (*γ* = 0.59) could be found for *DIAPH1*.

*RASGRP2* (MIM: 605577) was recently implicated in a new form of Glanzmann’s-like thrombasthenia (MIM: 273800) based on data from a single pedigree.^33^ Glanzmann’s is characterized by impaired platelet aggregation, leading to excessive bleeding. Under a recessive mode of inheritance, our similarity regression successfully detects an association (*γ* = 0.75) for *RASGRP2* and estimates a characteristic phenotype concentrated around “Abnormal platelet aggregation” (Figure 4). It is characteristic of Glanzmann’s that platelet aggregation is impaired in response to multiple agonists because their common downstream effect—the binding of platelets to fibrinogen—is impeded by the presence of reduced numbers of fibrinogen receptors. Here we also observe this phenomenon but only collagen-induced platelet aggregation carries significant weight in the characteristic phenotype because it is the only specific aggregation term that is shared by all the cases of this recently discovered disorder. There is also a very low probability of inclusion of two rare terms that are not related to the disease—“Atypical scarring of skin” and “Intracranial meningioma”—because of a chance comorbidity in one of the affected cases.

### Overall Results

**Figure 5:**
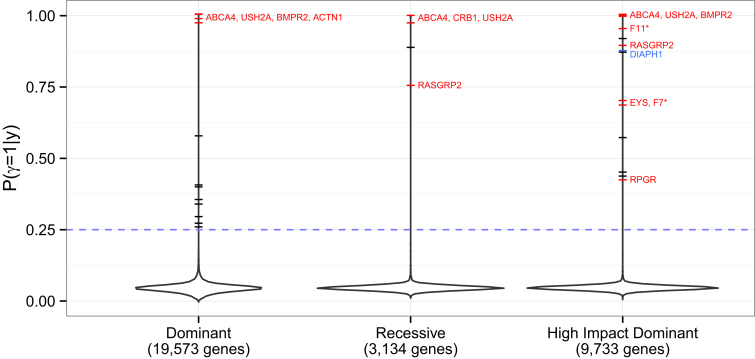
Overall Results Distributions of the estimated posterior means of γ obtained by applying the SimReg method to each gene under three different modes of inheritance. The tails are truncated at the most extreme values. The dashes indicate values greater than 0.25. The known genes for the BRIDGE project disorders having ℙ(γ=1|y)>0.25 and a compatible inferred phenotype are labeled and colored in red. An asterisk indicates that a posterior mean of *γ* greater than 0.25 was estimated only with the use of a prior on *ϕ* that was informed by the literature of human and murine heritable disorders.

Finally, we turn our attention to the overall results of applying the inference procedure to data for all genes under the three modes of inheritance considered, subject to $\sum_{i}y_{i}\geq 2$. In total, we applied the inference to 19,573, 3,134 and 9,733 genes for the dominant, recessive, and high-impact dominant modes of inheritance, respectively. The estimates of ℙ(γ=1|y) are shown as vertical density plots in Figure 5. For the majority of genes (65%), ℙ(γ=1|y)<ℙ(γ=1)=0.05, which implies that no characteristic phenotype can be found that helps distinguish carriers of the rare genotype from other subjects. This result is consistent with the expectation that variants in only a small proportion of genes are implicated in these rare diseases and indicates that specificity is largely controlled.

Strikingly, under all three assumed modes of inheritance, most of the highly confident results (i.e., the genes for which the estimates of ℙ(γ=1|y) are close to one) are for genes known to be relevant to the pathologies of the patients (indicated by red labels in Figure 5). In all but one case (*KIF1A* [MIM: 601255]), where a gene had ℙ(γ=1|y)>0.25 and was in one of the projects’ set of known genes, a characteristic phenotype similar to the known phenotype was inferred (Table 2). Above a threshold of ℙ(γ=1|y)=0.25, there was a significant enrichment for known genes (Fisher exact test p = 2.39 × 10^−4^, 1.98 × 10^−4^, and 2.23 × 10^−7^ for the dominant, recessive, and high-impact dominant modes of inheritance, respectively). Some of the inferred known genes are highlighted more than once across the three modes of inheritance in Figure 5 because there is power to detect the association even when the mode of inheritance is misspecified. For example, *RASGRP2*-related Glanzmann’s is recessive, yet ℙ(γ=1|y)>0.25 even if a high-impact dominant mode of inheritance is assumed.

The black dashes in Figure 5 correspond to unknown genes for which the inferred ℙ(γ=1) is greater than 0.25, of which there were 8, 1, and 5 found for the dominant, recessive, and high-impact dominant model of inheritance, respectively. These candidates are genes with potentially novel roles in disease and are being actively explored.

## Discussion

We have described a method for identifying the genetic determinants of rare diseases that does not require the disease phenotype to be specified a priori. The method uncovers associations between rare genotypes and the similarities between subject phenotypes and a latent characteristic phenotype. Throughout this paper, rare variants have been aggregated within genes according to a hypothesized mode of inheritance in order to define presence or absence of a rare genotype. However, the unit of analysis could be a set of interacting domains or any other arbitrary genomic grouping. During final review of this work, a prioritization procedure was proposed that combines a standard measure of strength of phenotypic clustering among individuals having two loss-of-function variants in a gene and the probability of the variants appearing in opposite haplotypes in an outbred population.^34^ In contrast, our inference procedure is based on statistical principles and the formulation of a model that is flexible with regards to phenotypic expressivity and genetic architecture and robust to noisy clinical coding and moderate genetic heterogeneity. Our Bayesian model naturally accounts for prior evidence of disease phenotypes associated with variants in particular genes by differentially weighting the prior probability of inclusion of HPO terms in the characteristic phenotype. Our finding that variants in *DIAPH1* can cause macrothrombocytopenia is an example of how this up-weighting can improve the inference.

The approach we have described is a natural and powerful way of modeling many rare disease phenotypes because it accounts for phenotypic abnormalities across all organ systems encoded with variable precision. Studies of syndromic diseases in particular can benefit from this way of uncovering associations. Our model can also be used for predicting the log odds of the rare genotype using solely phenotype data by means of a function implemented in our SimReg software. This could be used to aid diagnosis by indicating which of a patient’s genes should be prioritized for sequencing based on his or her HPO terms. Finally, our regression approach might prove useful for performing inference using notions of similarity between terms in other ontologies where a binary response can be encoded.

Although our method improves significantly on modeling of phenotypic heterogeneity, our treatment of genetic heterogeneity can still be refined, because we currently rely on aggregation of genetic information into single binary variables. In the future we will explore improved modeling of genetic heterogeneity, in which the possibility of a mixture of pathogenic and neutral variants is accounted for explicitly. This would be applicable to genes in which different variants can cause drastically different clinical pathologies (e.g., *LMNA* [MIM: 150330]). Allele frequency, conservation, and functional information could also be used to modulate prior distributions.

In summary, our work represents an advancement in the statistical modeling of ontological heterogeneity that might prove useful at a time in which large collections of deeply phenotyped and sequenced cases are being assembled to uncover hitherto elusive causes of rare heterogeneous diseases.

## Figures and Tables

**Table 1:** Studies from which Genetic and Phenotypic Data Were Obtained

**Study**	**Phenotype**	**Unrelated Subjects**	**Known Genes**
Bleeding and Platelet Disorders (BPD)	detailed patient-specific HPO terms	709	74
Primary ImmunoDeficiency (PID)	Abnormality of the immune system (HP:0002715)	201	131
Pulmonary Arterial Hypertension (PAH)	Pulmonary hypertension (HP:0002092)	422	9
Specialist Pathology Evaluating Exomes in Diagnostics (SPEED)	Retinal dystrophy (HP:0000556)	384	241
Abnormality of the nervous system (HP:0000707)	215	689
Abnormality of the nervous system and Retinal dystrophy (HP:0000707, HP:0000556)	7	
Phenotypic abnormality (HP:0000118)	107	

Note that the SPEED project has a branch dealing with retinal dystrophy and another branch dealing with abnormalities of the nervous system and that 7 individuals are included in both branches. In addition, 107 subjects could not be assigned to a specific sub-project at the time of writing due to lack of information and we assigned them a single abstract HPO term “Phenotypic abnormality” (HP:0000118).

**Table 2:** Known Genes for which ℙ(γ=1|y)>0.25 and the Inferred Phenotype Was Compatible with the Known Disorder

**Gene**	**MIM No.**	**Mode of Inheritance**	**Known Disorder**	ℙ(γ=1|y)	**Highest Marginal Posterior Probability Terms in *ϕ***
*ACTN1*	102575	dominant	bleeding and platelet disorder	1.00	increased mean platelet volume (0.79), thrombocytopenia (0.56), platelet count (0.44)
*BMPR2*	600799	dominant	pulmonary arterial hypertension	1.00	pulmonary hypertension (0.34), elevated pulmonary artery pressure (0.31), pulmonary artery (0.11)
*ABCA4*	601691	recessive	retinal dystrophy	0.99	retinal dystrophy (0.22), retina (0.22), fundus (0.16)
*USH2A*	608400	recessive	retinal dystrophy	0.99	retina (0.23), retinal dystrophy (0.2), fundus (0.17)
*CRB1*	604210	recessive	retinal dystrophy	0.97	retinal dystrophy (0.21), retina (0.18), fundus (0.18)
*F11*	264900	high-impact dominant	bleeding and platelet disorder	0.95	reduced factor XI activity (0.89), intrinsic pathway (0.11), platelet aggregation (0.07)
*RASGRP2*	605577	recessive	bleeding and platelet disorder	0.75	platelet aggregation (0.67), collagen-induced platelet aggregation (0.2), platelet function (0.1)
*EYS*	612424	high-impact dominant	retinal dystrophy	0.70	retinal dystrophy (0.2), retina (0.17), fundus (0.14)
*F7*	613878	high-impact dominant	bleeding and platelet disorder	0.68	extrinsic pathway (0.5), reduced factor vii activity (0.46), white hair (0.1)
*RPGR*	312610	high-impact dominant	retinal dystrophy	0.42	retina (0.2), retinal dystrophy (0.17), posterior segment of the eye (0.16)

We display the mode of inheritance under which the association was found, the known disorder, the probability of association, and the top three HPO terms (shown in abbreviated form) in the inferred phenotypes. The marginal posterior probability of inclusion in the characteristic phenotype is shown in brackets next to each term. When an association was found under multiple modes of inheritance, only the true mode is shown. Note that the inferred phenotypes are influenced by prior phenotypic information in the form of OMIM and MGI annotations.

## Web Resources

The URLs for data presented herein are as follows:

Care for Rare, http://care4rare.ca
Comprehensive R Archive Network, http://cran.r-project.org
ExAC Browser, http://exac.broadinstitute.org/
Genomics England, http://www.genomicsengland.co.uk
HPO, http://compbio.charite.de/jenkins/job/hpo/
Mouse Genome Informatics, http://www.informatics.jax.org/
NIHR BioResource – Rare Diseases, https://bioresource.nihr.ac.uk/rare-diseases/
OMIM, http://www.omim.org/
RetNet – Retinal Information Network, https://sph.uth.edu/retnet/home.htm
Undiagnosed Diseases Network, http://www.genome.gov/27550959

## Supplemental Data

Document S1. Supplemental Note

Table S1. Additional Members and Collaborators of the NIHR BioResource

Document S2. Article plus Supplemental Data
