# Supplementary material for: Phenotype Similarity Regression for Identifying the Genetic Determinants of Rare Diseases
Source: Am J Hum Genet. 2016 Feb 25;98(3):490–9. doi: 10.1016/j.ajhg.2016.01.008 (PMC4827100; doi:10.1016/j.ajhg.2016.01.008)
Supplement: Document S1. Supplemental Note [file mmc1.pdf]

The American Journal of Human Genetics, Volume *98*

Supplemental Information

Phenotype Similarity Regression for Identifying the Genetic Determinants of Rare Diseases

Daniel Greene, Sylvia Richardson, Ernest Turro, and NIHR BioResource

1 Diagram representing the $\gamma = 1$ model

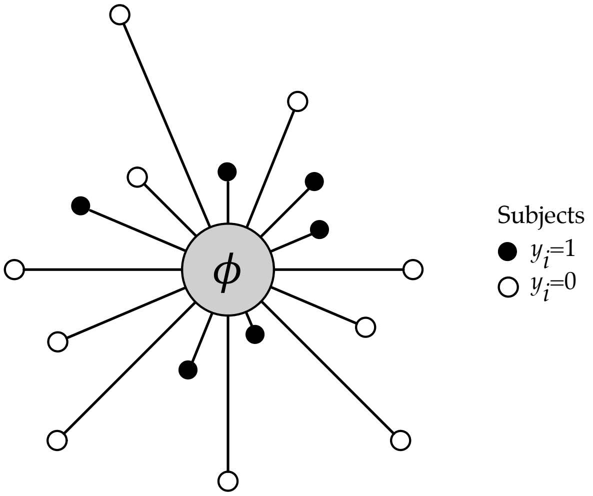

Figure S1: **Cartoon depicting the $\gamma = 1$ model.** Individuals are more likely to carry a rare genotype (indicated by a filled dot) if they are phenotypically similar (as indicated by short edges) to the characteristic phenotype ϕ than if they are dissimilar to it (as indicated by long edges). The angular directions of the edges are purely representational and should not be interpreted. In contrast, under $\gamma = 0$, the rare genotype occurs at a fixed rate irrespective of phenotype.

2 Detailed model specification

The full specification of the two alternative models, $\gamma = 0$ and $\gamma = 1$, described in the main text is given below.

$\gamma = 0$

$$\begin{aligned} y_i &\sim \text{Bernoulli}(p_i), \\ \log\left(\frac{p_i}{1-p_i}\right) &= \alpha + \hat{h}_i, \end{aligned}$$

with

$$\alpha \sim \text{Normal}(\mu_\alpha, \sigma_\alpha^2),$$

and where $\hat{h}_i$ is an optional plug-in offset parameter (see Section 3).

$\gamma = 1$

$$\begin{aligned} y_i &\sim \text{Bernoulli}(p_i), \\ \log\left(\frac{p_i}{1-p_i}\right) &= \alpha + \beta f\left(S_\phi(\phi \to x_i), a_f, b_f\right) \cdot g\left(S_x(x_i \to \phi), a_g, b_g\right) + \hat{h}_i, \end{aligned}$$

with

$$
\begin{aligned}
\alpha &\sim \text{Normal}(\mu_\alpha, \sigma_\alpha^2), \\
\log(\beta) &\sim \text{Normal}(\mu_\beta, \sigma_\beta^2), \\
\log \frac{a_f}{b_f} &\sim \text{Normal}(\mu_f, \sigma_f^2), \\
\log(a_f + b_f) &\sim \text{Normal}(\mu_{f'}, \sigma_{f'}^2), \\
\log \frac{a_g}{b_g} &\sim \text{Normal}(\mu_g, \sigma_g^2), \\
\log(a_g + b_g) &\sim \text{Normal}(\mu_{g'}, \sigma_{g'}^2), \\
\mathbb{P}(\phi) &= \begin{cases} \frac{1}{|\Phi^{(k)}|} & \text{No literature phenotype} \\ \frac{S'(M \to \phi)}{\sum\limits_{\psi \in \Phi^{(k)}} S'(M \to \psi)} & \text{Literature phenotype } M \end{cases}
\end{aligned}
$$

where $\Phi^{(k)}$ is the set of all minimal sets of HPO terms of size k. We use the following definitions of S_ϕ, S_x, f, g, S':

$$
\begin{aligned}
S_\phi(\phi \to x_i) &= \frac{1}{|\phi|} \sum_{t_\phi \in \phi} \max_{t_x \in x_i} s(t_\phi, t_x) \mathbb{1}_{t_\phi \in \text{anc}(t_x)}, \\
S_x(x_i \to \phi) &= \frac{1}{|x_i|} \sum_{t_x \in x_i} \max_{t_\phi \in \phi} s(t_x, t_\phi) \mathbb{1}_{t_\phi \in \text{anc}(t_x)}, \\
f(z, a_f, b_f) &= I_z(a_f, b_f), \\
g(z, a_g, b_g) &= I_z(a_g, b_g), \\
S'(M \to \tau) &= \frac{1}{|\tau|} \sum_{t \in \tau} \max_{m \in M} \exp\left(s'(m, t)\right),
\end{aligned}
$$

with

$$
\begin{aligned}
s'(t_1, t_2) &= \max_{t \in \text{anc}(t_1) \cap \text{anc}(t_2)} \text{IC}(t), \\
s(t_1, t_2) &= \frac{2 \times s'(t_1, t_2)}{\text{IC}(t_1) + \text{IC}(t_2)}, \\
I_z(a, b) &= \frac{\int_0^z t^{a-1}(1-t)^{b-1} \text{d}t}{\int_0^1 t^{a-1}(1-t)^{b-1} \text{d}t}
\end{aligned}
$$

and where anc(t) is the union of t and all the ancestors of t in the HPO graph and IC(t) is the information content of term t. Finally, we use the following values for the hyperparameters (see also Section 4):

$$
\mu_\alpha = 0,\ \sigma_\alpha^2 = 5,\ \mu_\beta = 2,\ \sigma_\beta^2 = 1,\ \mu_f = 1,\ \sigma_f^2 = 1,\ \mu_{f'} = 2,\ \sigma_{f'}^2 = 1,\ \mu_g = 0,\ \sigma_g^2 = 1.5,\ \mu_{g'} = 2,\ \sigma_{g'}^2 = 1 \quad \text{(S1)}
$$

3 Estimation of the offset $\hat{h}_i$

In order to accommodate prior beliefs about the background rate of observing the rare genotype for a particular gene, we obtained point estimates of the effects of gene length and sequencing platform on the log odds of observing the rare genotype. We fitted a generalised linear model linking these variables to the genotype data across all genes for all 2,045 sequenced individuals described in the main text. The model used was:

$$
\begin{aligned}
y_{ij} &\sim \text{Bernoulli}(p_{ij}), \\
\log\left(\frac{p_{ij}}{1 - p_{ij}}\right) &= \lambda l_j + \omega^T z_{i\cdot},
\end{aligned}
$$

where $y_{ij} = 1$ indicates presence of the rare genotype in gene j for individual i, which occurs with probability p_{ij}, l_j is the length in base pairs of the coding region of gene j and $z_{ik} = 1$ if individual i was sequenced on sequencing platform k and 0 otherwise. Thus, λ is interpretable as the effect size of gene length and $\omega_1, \ldots, \omega_K$ as the effect

sizes of sequencing platforms $1, \ldots, K$. We found that certain sequencing platforms led to gene-specific biases in variant calls. To ensure robustness to these biases, we only used data for genes having a Fisher exact p-value of association between the rare genotype and the sequencing platform greater than 0.05. Under a model of no association, the offset for gene j is given by:

$$y_i \sim \text{Bernoulli}(p_i),$$
$$\log\left(\frac{p_i}{1-p_i}\right) = \alpha + \hat{h}_i,$$

where $\hat{h}_i = \hat{\lambda} l_j + \hat{\omega}^T z_{i\cdot}$. The $\hat{h}_i$ was obtained for all genes in all the hypothetical modes of inheritance described in the main text.

4 Prior on f and g

Recall that the overall predictor for the log odds of having a rare genotype under the alternate model is given by

$$f(S_\phi(\phi \to x_i), a_f, b_f) \cdot g(S_x(x_i \to \phi), a_g, b_g).$$

As described in the main text, the presence of a term in the characteristic phenotype ϕ that is absent from the patient phenotype x_i has the effect of lowering S_ϕ, while the presence of a term in x_i that is absent from ϕ has the effect of lowering S_x. For example, if ϕ has one HPO term and it is also present in x_i, then $S_\phi = 1$. However, the presence of one or two additional spurious terms can reduce S_ϕ to as low as 0.5 or 0.33 respectively.

In order to discourage non-parsimonious characteristic phenotypes, we place a high prior weight on f transformations whose corresponding probability density functions have means above 0.5 (i.e. $\frac{a_f}{a_f+b_f} > 0.5$) as this ensures that a good prediction of the log odds cannot be obtained if the absolute value of S_ϕ is low. Specifically, we specify the priors on the parameters of f described in Section 2. The resultant distribution of transformations f and g are represented in Figure S2 (left). However, in order to allow for patients coded with sporadic terms that are not part of the core disease phenotype, we specify a more flexible prior distribution on g than we do on f, as illustrated in Figure S2 (right).

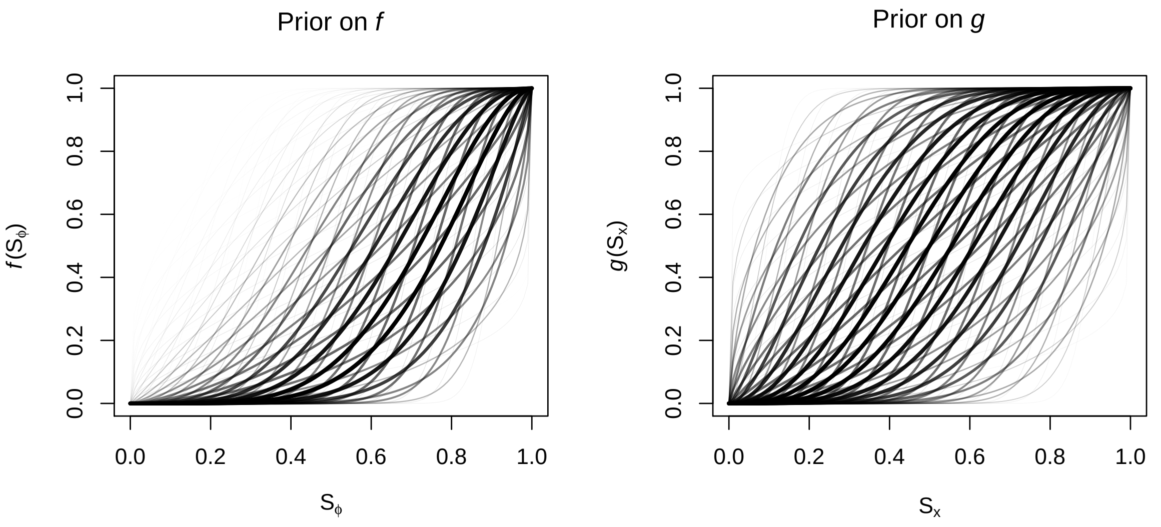

Figure S2: **Prior on f and g.** We show the distribution of shapes for the incomplete beta function transformation of phenotype similarities S_ϕ and S_x for select values of the parameters, given the hyperparameter values in Equation S1. The thickness and opacity of each line is proportional to the prior probability of the corresponding parameterisation of the transformation.

Our choice of hyperparameter values was informed by a sensitivity analysis assessing the model's performance on data for *ACTN1*. We found that not using a transformation at all (i.e. not modulating the similarity with f and

g, which is equivalent to using the identity function obtained by setting $a_f = b_f = a_g = b_g = 1$), or using an overly flexible prior on f, discourages inclusion of the essential 'Thrombocytopenia' term relative to inclusion of spurious alternative terms, conditional on inclusion of the other essential term, 'Increased mean platelet volume'. This occurs because if the value of $\frac{a_f}{a_f+b_f}$ has high posterior weight near 0.5, then spurious terms can be accommodated by mapping values near 0.5 to near 1. As more prior weight is shifted to f transformations with a value of $\frac{a_f}{a_f+b_f}$ greater than 0.5, the probability of joint inclusion of the two key nodes of this disease is increased (Figure S3).

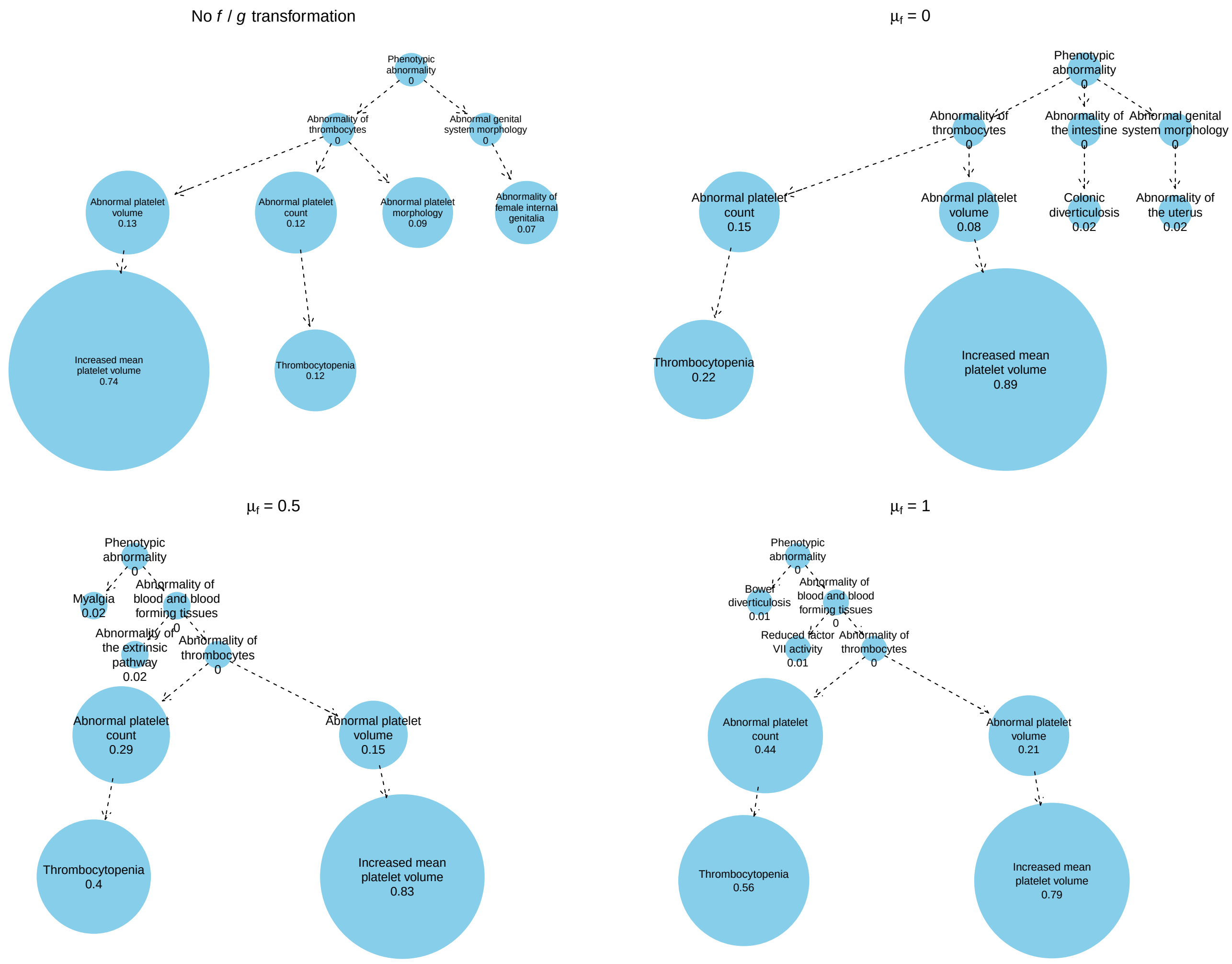

Figure S3: **Inferred ϕ for various parameterisations of the similarity function.** Graphical representation of the posterior distribution of ϕ when no f/g transformations are used and for different values of μ_f (with $\sigma_f^2 = 1, \mu_g = 0, \sigma_g^2 = 1.5$) using the data for *ACTN1*. Each node shows the marginal probability of inclusion in ϕ. Without the f/g transformations, the essential 'Thrombocytopenia' term carries low posterior weight. If the f/g transformations are included, as the value of μ_f is increased, from 0 through 0.5 to 1, the probability of inclusion of the term 'Thrombocytopenia' increases.

Our choice of prior can nevertheless accommodate sporadic absence of disease terms in patients that are part of the characteristic phenotype, provided it can be estimated accurately. Our simulation study (see main text) confirms this because we observe a gradual reduction of the posterior mean value of $\frac{a_f}{a_f+b_f}$ as the expressivity of the terms of the template phenotype for the hypothetical disease phenotype decreases from 1 through $\frac{2}{3}$ to $\frac{1}{3}$ (Figure S4).

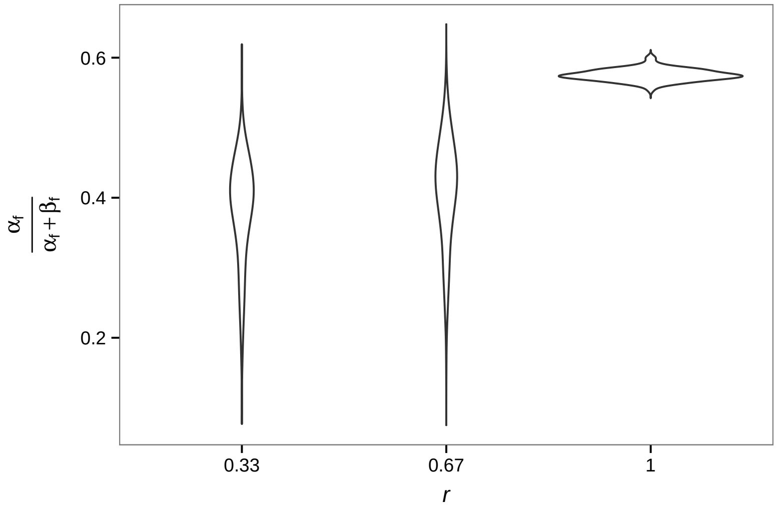

Figure S4: **The posterior mean value of $\frac{a_f}{a_f+b_f}$ for different levels of expressivity.** Distribution of the posterior mean value of $\frac{a_f}{a_f+b_f}$ for three different values of expressivity r. The distributions were obtained over 384 repetitions of our simulation, with $\sum_i y_i = 20$. A decrease in the expressivity, r, of individual terms in the template phenotype results in a decrease in the posterior mean value of $\frac{a_f}{a_f+b_f}$.

5 Genetic heterogeneity

We performed a different version of the simulation study in the main text to assess the performance of our method when genetic heterogeneity is controlled explicitly. Here, we vary a parameter representing the extent of genetic heterogeneity, v, so that for each individual having $y_i = 1$, there were v additional individuals with phenotypes simulated from the same distribution but having $y_i = 0$.

We applied the inference to data sets generated with $v \in \{0, 1, 3, 9\}$. Thus, the simulations where $v = 0$ correspond to the scenario of the simulations described in the main text, and those where $v = 9$ represent situations where only one tenth of the cases having a phenotype arising from the disease template have $y_i = 1$.

The results of the simulation, given as box plots of the estimated posterior means of γ under the various scenarios (Figure S5), demonstrate that although power goes down as genetic heterogeneity increases, the sensitivity of the method, thresholding on $\gamma > 0.25$, approaches 100% when expressivity r is 1 and $\sum_i y_i$ is at least 6, and also when expressivity r is $\frac{2}{3}$ and $\sum_i y_i$ is at least 10, irrespective of v. When $v = 3$ and $r = \frac{1}{3}$, which means the HPO terms have very low expressivity and only a quarter of individuals drawn from the template phenotype carry the rare genotype, γ exceeded 0.25 in 87.5% of our simulations as long as 20 out of $1,000$ individuals carried the genotype. Thus we conclude that our method is powerful even in challenging scenarios in which there is substantial genetic heterogeneity and low phenotypic expressivity.

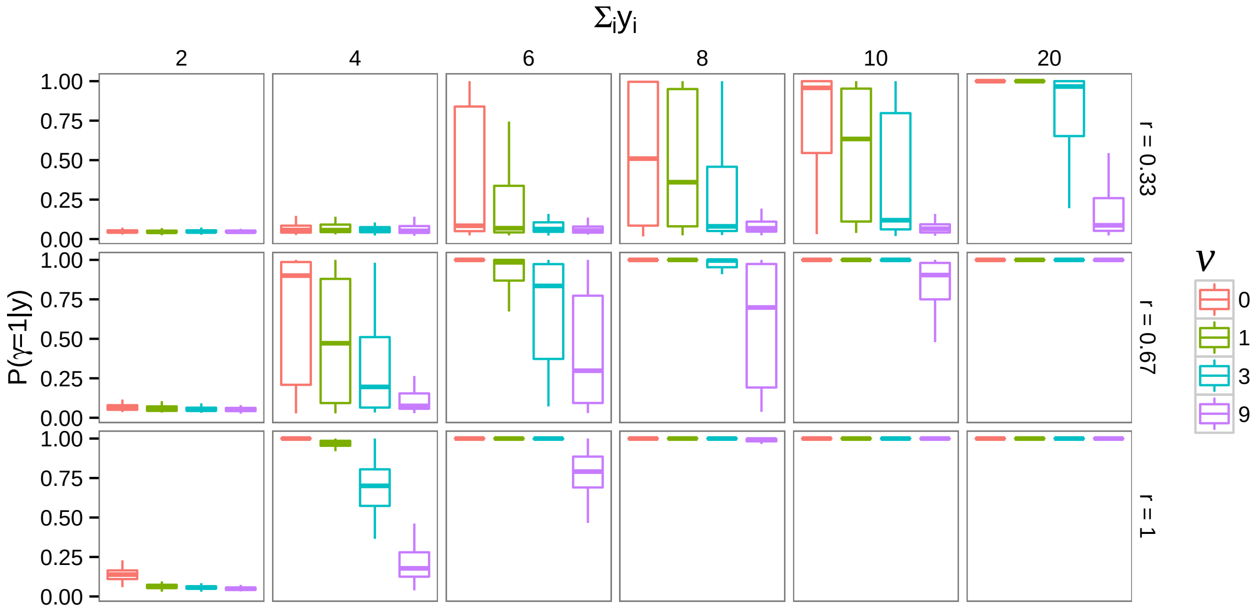

Figure S5: **Relationship between genetic heterogeneity and power.** Box plots showing the distribution of the posterior estimates of γ at various levels of phenotypic expressivity, r, for various sample frequencies of the rare genotype, $\sum_i y_i$ and with different levels of genetic heterogeneity as captured by v. The boxes contain the inter-quartile range, with whiskers extending to the extreme values up to 1.5 times the inter-quartile range from the box.

6 Specificity

The simulation study presented in the main text shows that if the phenotypes are homogeneously selected from a wide range of HPO nodes, then our method is unlikely to produce high posterior estimates of γ. However, the simulation in the main text is based on only 64 repetitions for each simulation set-up (shown as 64 grey dots in each panel). In order to more accurately assess the specificity of our method we simulated 20,000 independent sets of phenotypes, simulated with a total of 6 cases having the rare genotype. The distribution of the posterior mean values of γ inferred for the data sets are shown in the left panel of Figure S6. There were a total of 7 simulated data sets for which the value was greater than 0.25, with the highest estimate being equal to 0.86, which equates to a specificity of 99.97%. The data set for which the highest value was obtained contained four (out of six) individuals with the rare genotype, labelled, 3–6, who had a high mean posterior similarity (> 0.3) to the characteristic phenotype (middle panel of Figure S6). By chance, these four individuals had been assigned highly specific terms relating to bone ossification, the toe and long bone morphology (right panel of Figure S6). This coincidental sharing of HPO terms by these individuals who also carried the rare genotype led to the abnormally high posterior estimate of γ. However, this is a desirable property of our method because in practice it is not possible to know whether such a correlation is causal or spurious.

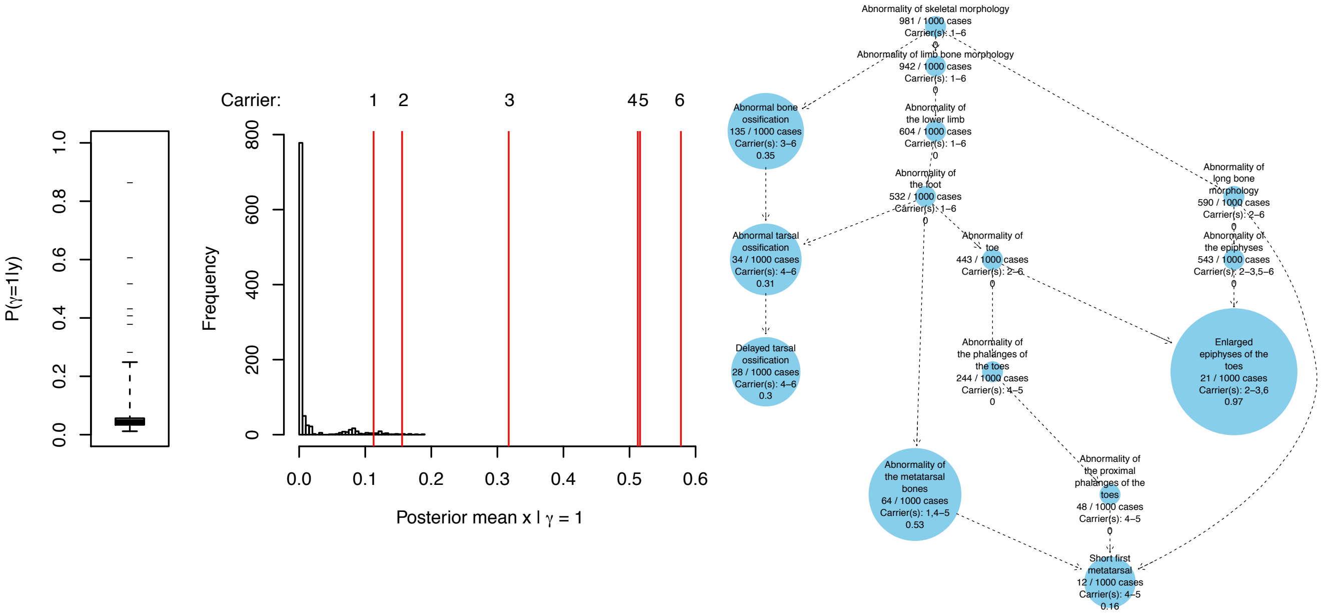

Figure S6: **Evaluation of the specificity of the inference procedure.** The distribution of posterior γ for applications of the inference to the 20,000 repeats of the simulation is shown as a box plot. The box contains the inter-quartile range, with whiskers extending between the lowest posterior γ obtained and 0.25. For the simulated data set for which the highest posterior mean value of γ was inferred, the posterior mean similarities to ϕ, x_i, for the 1,000 simulated patient phenotypes are shown as a histogram, with those of the individuals with the rare genotype, $i|y_i = 1$, marked by red lines. The inferred characteristic phenotype ϕ for this data set is shown as a graph. Each node is labelled with a) the HPO term b) the number of simulated individuals out of 1,000 who had the term c) which individuals with the rare genotype had the term (as labelled in the middle panel) and d) the posterior probability of inclusion in ϕ conditional on $\gamma = 1$ (also represented by node size).

7 Inference using Markov chain Monte Carlo (MCMC)

7.1 Carlin and Chib method

The method of Carlin and Chib is a means of inferring the parameters in two models and computing a Bayes factor comparing them. Instead of targeting the posterior distribution of each model individually, the following function is targeted:

$$(\gamma, \theta^{(0)}, \theta^{(1)}) \mapsto (1-\gamma)L_y^{(0)}(\theta^{(0)})p_0(\theta^{(0)})f_1(\theta^{(1)}) + \gamma L_y^{(1)}(\theta^{(1)})p_1(\theta^{(1)})f_0(\theta^{(0)}).$$

Here, $\theta^{(0)}$ and $\theta^{(1)}$ are vectors of the parameters of models 0 and 1 respectively and $p_0(\theta^{(0)})$ and $p_1(\theta^{(1)})$ are their respective priors. The likelihood functions under model 0 and 1 are given by $L_y^{(0)}(\theta^{(0)})$ and $L_y^{(1)}(\theta^{(1)})$ respectively. The functions $f_0(\theta^{(0)})$ and $f_1(\theta^{(1)})$ are arbitrary probability density functions called 'pseudopriors' representing the conditional probability distributions of the parameters of one model given the alternate model is true, i.e. $\theta^{(0)}|\gamma = 1$ and $\theta^{(1)}|\gamma = 0$ respectively. The conditional posterior distributions of the parameters $\theta^{(0)}|\gamma = 0$ and $\theta^{(1)}|\gamma = 1$ can be estimated from MCMC samples made at iterations when $\gamma = 0$ and $\gamma = 1$, respectively, and the posterior probability that model 1 is true can be estimated from the proportion of iterations in which $\gamma = 1$.

Let α^* be the intercept parameter under $\gamma = 0$ and α be the intercept parameter under $\gamma = 1$ so that they may be distinguished. For convenience, we perform inference of $\tilde{\phi}$, which is on the unrestricted space of vectors of HPO terms, rather than ϕ, because it is difficult to propose uniformly from the space of minimal sets $\Phi^{(k)}$. However, ϕ can be recovered from $\tilde{\phi}$ easily by mapping to the corresponding minimal set. The MCMC algorithm proceeds to target the following distribution:

$$\begin{aligned} &\mathbb{P}(\gamma, \alpha, \beta, a_f, b_f, a_g, b_g, \tilde{\phi}|y) \propto \\ (1-\gamma)&L_y^{(0)}(\alpha^*)p_0(\alpha^*)f_1(\alpha)f_1(\beta)f_1(a_f)f_1(b_f)f_1(a_g)f_1(b_g)f_1(\tilde{\phi}) \\ &+\gamma L_y^{(1)}(\alpha, \beta, a_f, b_f, a_g, b_g, \tilde{\phi})p(\alpha)p(\beta)p(a_f)p(b_f)p(a_g)p(b_g)p(\tilde{\phi})f_0(\alpha^*). \end{aligned}$$

For optimal mixing of the Markov chain, the pseudopriors should approximate the respective conditional posterior distribution given the model, that is, $f_0(\theta^{(0)}) \propto L_y^{(0)}(\theta^{(0)})p_0(\theta^{(0)})$ and $f_1(\theta^{(1)}) \propto L_y^{(1)}(\theta^{(1)})p_1(\theta^{(1)})$. To achieve this, we tune the pseudopriors using empirical summary statistics obtained by running initial Markov chains under each model separately. For parameters α^* and α, Normal pseudopriors are used, while for the strictly positive parameters β, a_f, b_f, a_g and b_g, Log-Normal pseudpriors are used. The hyperparameters of these pseudopriors are obtained using maximum likelihood estimation based on the MCMC samples. We compose a pseudoprior for $\tilde{\phi}$ by counting the number of appearances of HPO terms in any of the k slots of $\tilde{\phi}$ throughout the tuning iterations:

$$\mathbb{P}(t) = \frac{\sum_{i=1}^{I}\sum_{j=1}^{k} \mathbb{1}(\tilde{\phi}_{ij} = t) + \epsilon}{Ik + \epsilon|H|}, \tag{S2}$$

where I is the number of MCMC tuning iterations, t is a term in the set of HPO terms H and $\tilde{\phi}_{ij}$ is the jth element of $\tilde{\phi}$ in the ith iteration. We allow a non-zero probability of inclusion of terms which have not been sampled at all during the tuning batch by setting $\epsilon = 1$. Using the above expression, we define the pseudoprior on $\tilde{\phi}$ as

$$f_1\left(\tilde{\phi}\right) = \prod_{j=1}^{k} \mathbb{P}\left(\tilde{\phi}_j\right).$$

7.2 MCMC updates

Each iteration of the MCMC algorithm comprises the following steps:

1. An update of α^*:

   $\gamma = 0$ Propose an update of α^* by drawing from

   $$\alpha^{*'} \sim \text{Normal}(\alpha^*, s_\alpha^2)$$

   and accepting with probability

   $$\min\left(1, \frac{L_y^{(0)}(\alpha^{*'})p(\alpha^{*'})}{L_y^{(0)}(\alpha^*)p(\alpha^*)}\right).$$

   $\gamma = 1$ Sample $\alpha^{*'}$ from the pseudoprior distribution for α^*:

   $$\alpha^{*'} \sim \text{Normal}(\hat{\mu}_{\alpha^*}, \text{sd} = \hat{\sigma}_{\alpha^*}^2).$$

2. An update of α:

   $\gamma = 0$ Sample α' from the pseudoprior distribution for α:

   $$\alpha' \sim \text{Normal}(\hat{\mu}_\alpha, \hat{\sigma}_\alpha^2).$$

   $\gamma = 1$ Propose an update of α by drawing from

   $$\alpha' \sim \text{Normal}(\alpha, s_\alpha^2)$$

   and accepting with probability

   $$\min\left(1, \frac{L_y^{(1)}(\alpha', \beta, a_f, b_f, a_g, b_g, \tilde{\phi})p(\alpha')}{L_y^{(1)}(\alpha, \beta, a_f, b_f, a_g, b_g, \tilde{\phi})p(\alpha)}\right).$$

3. An update of β:

   $\gamma = 0$ Sample $\log \beta'$ from the pseudoprior distribution for $\log \beta$

   $$\log \beta' \sim \text{Normal}(\hat{\mu}_\beta, \hat{\sigma}^2_\beta).$$

   $\gamma = 1$ Propose an update of $\log \beta$ by drawing from

   $$\log \beta' \sim \text{Normal}(\beta, s^2_\beta)$$

   and accepting with probability

   $$\min\left(1, \frac{L^{(1)}_y(\alpha, \beta', a_f, b_f, a_g, b_g, \tilde{\phi})p(\beta')}{L^{(1)}_y(\alpha, \beta, a_f, b_f, a_g, b_g, \tilde{\phi})p(\beta)}\right).$$

4. An update of the shape parameters a_f, b_f, a_g and b_g analagously as is done for β.

5. An update of $\tilde{\phi}$:

   $\gamma = 0$ Sample $\tilde{\phi}$ from the pseudoprior distribution for $\tilde{\phi}$ by sampling all k terms independently from the distribution described in Equation S2.

   $\gamma = 1$ Propose updating $\tilde{\phi}$ to $\tilde{\phi}'$ by setting component $t = \left[\tilde{\phi}\right]_j$ (where j is chosen at random from $1, \ldots, k$) to a random term t', selected with probability π_t. Hence $\tilde{\phi}'$ can be specified as

   $$\left[\tilde{\phi}'\right]_h = \begin{cases} t' & h = j \\ \left[\tilde{\phi}\right]_h & \text{otherwise.} \end{cases}$$

   The proposal is accepted with probability

   $$\min\left(1, \frac{L^{(1)}_y(y|\alpha, \beta, a_f, b_f, a_g, b_g, \tilde{\phi}')p(\tilde{\phi}')\pi_t}{L^{(1)}_y(\alpha, \beta, a_f, b_f, a_g, b_g, \tilde{\phi})p(\tilde{\phi})\pi_{t'}}\right).$$

   We set the proposal distribution of the new term $\{\pi_t : t \in \mathrm{H}\}$ to equal that of the individual components of $\tilde{\phi}$ under its pseudoprior (see Equation S2). An alternative approach that does not rely on a tuning chain is to propose a new term proportionally to the number of subjects having $y_i = 1$ whose phenotypes include the term or one of its descendants in the HPO:

   $$\pi_t \propto \sum_{i=1}^{N} \mathbb{1}_{y_i=1} \mathbb{1}_{t \in \bigcup_{t' \in x_i} \text{anc}(t')}.$$

6. An update of γ by Gibbs sampling:

   $$\gamma' \sim \text{Bernoulli}\left(\frac{\omega^{(1)}}{\omega^{(0)} + \omega^{(1)}}\right),$$

   where

   $$\begin{aligned} \omega^{(0)} &= (1-\pi)\, L^{(0)}_y(\alpha^*) f_1(\alpha) f_1(\beta) f_1(a_f) f_1(b_f) f_1(a_g) f_1(b_g) f_1(\tilde{\phi}) p(\alpha^*), \\ \omega^{(1)} &= \pi\, L^{(1)}_y(\alpha, \beta, a_f, b_f, a_g, b_g, \tilde{\phi}) p(\alpha) p(\beta) p(a_f) p(b_f) p(a_g) p(b_g) p(\tilde{\phi}) f_0(\alpha^*), \end{aligned}$$

   where π is the prior probability that $\gamma = 1$.

8 Calculation of prior probability for ϕ and $\tilde{\phi}$

In order to calculate $p(\phi)$ when using a uniform distribution over $\Phi^{(k)}$, we need to calculate the number of distinct minimal sets $\left|\Phi^{(k)}\right|$. This is trivial when $k = 1$, as $\left|\Phi^{(k)}\right| = |\mathrm{H}|$. However it becomes more computationally intensive as k increases, so in our implementation we use the approximation $\binom{|\mathrm{H}|}{k}$. This approximation works well in practice when k is small. It has no effect on the update of the $\tilde{\phi}$ parameter, as the $\left|\Phi^{(k)}\right| = |\mathrm{H}|$ expression cancels out in the acceptance probability for $\tilde{\phi}'$, but it does affect the update of γ as it penalises the model $\gamma = 1$ slightly by overestimating the size of $\left|\Phi^{(k)}\right|$.

When using an informative prior distribution, weighted by similarity to the literature phenotype as described in the main text, we need to calculate $\sum_{\psi \in \Phi^{(k)}} S'(M \to \psi)$. In order to avoid having to sum over the entire space $\Phi^{(k)}$, we employ the approximation $\left|\Phi^{(k)}\right| \times k \times \mathrm{mean}_{\psi \in \mathrm{H}} S'(M \to \psi)$.

Finally, to compute $p(\tilde{\phi})$, we also need to calculate the number of alternative unrestricted vectors that map to the same minimal set, i.e. $\left|\left\{\tilde{\phi}' \in \mathrm{H}^k : \upsilon(\tilde{\phi}') = \upsilon(\tilde{\phi})\right\}\right|$, where ν maps an unrestricted vector to a minimal set. We use the following expression for the number of representations:

$$\left|\bigcup_{t \in \upsilon(\tilde{\phi})} \mathrm{anc}(t)\right|^k + \sum_{i=1}^{|\upsilon(\tilde{\phi})|} (-1)^i \binom{|\upsilon(\tilde{\phi})|}{i} \left(\left|\bigcup_{t \in \upsilon(\tilde{\phi})} \mathrm{anc}(t)\right| - i\right)^k$$

9 Ethics

Table S1 lists the ethics authorities for which the NIHR BioResource – Rare Diseases study has approval. All study procedures were performed after the participants provided informed written consent and were in accordance with the Declaration of Helsinki.

Name of national ethics authority	Ethics approval number	Country
Cambridgeshire 1 Research Ethics Committee	10/H0304/66	United Kingdom
East of England – Cambridge Central	13/EE/0325	United Kingdom
Institut National de La Santé et de la Recherche Médicale	RBM-01-14	France
Sir Charles Gairdner Group Human Research Ethics Committee	2012-095	Australia
Ethics Committee of the University Hospital Leuven	ML3580	Belgium
Ethics Board of the University of Greifswald	n/a	Germany
Ethics Board 2 at Campus Virchow – Klinikum, Charité University Hospital, Berlin	EA2/170/05	Germany
Children's Hospital of Philadelphia Institutional Review Board	IRB#12-008603	USA
Beth Israel Deaconess Medical Center IRB	Protocol #: 2011P000337	USA

Table S1: **Ethics approval information.** Names, ethics approval numbers and countries of ethics authorities approving the NIHR BioResource – Rare Diseases study.

10 SimReg performance

We applied the inference procedure to simulated data sets to assess the performance of SimReg. We varied the number of phenotyped individuals and the number of terms (sampled from a preset collection of approximately 1,000 terms) allocated to each individual, and programmed the algorithm to generate 20,000 MCMC samples (of which 10,000 are tuning iterations). The results of the performance test are shown in Table S2.

N	2 terms	4 terms	8 terms
100	7.31	9.03	11.55
1,000	47.38	65.70	91.74
10,000	451.54	627.75	879.78

Table S2: **Computational performance.** Completion times in seconds for applications of the SimReg procedure. The rows indicate the total number of individuals included in the inference, and the columns indicate the number of HPO phenotype terms allocated to each individual. These results were obtained by running SimReg on a single CPU of a computer with 2.40GHz processors.

11 Lists of known genes for the BRIDGE projects

Genes for which variants are known to underlie a BRIDGE project disorder are listed below:

Bleeding and Platelet Disorders (BPD)
ACTN1, *ANKRD26*, *ANO6*, *AP3B1*, *BLOC1S3*, *BLOC1S6*, *CYCS*, *DTNBP1*, *ETV6*, *F10*, *F11*, *F13A1*, *F13B*, *F2*, *F5*, *F7*, *F8*, *F9*, *FERMT3*, *FGA*, *FGB*, *FGG*, *FLI1*, *FLNA*, *GATA1*, *GFI1B*, *GGCX*, *GNE*, *GP1BA*, *GP1BB*, *GP6*, *GP9*, *HOXA11*, *HPS1*, *HPS3*, *HPS4*, *HPS5*, *HPS6*, *HRG*, *ITGA2B*, *ITGB3*, *LMAN1*, *LYST*, *MCFD2*, *MPL*, *MYH9*, *NBEA*, *NBEAL2*, *ORAI1*, *P2RY12*, *PLA2G4A*, *PLAT*, *PLAU*, *PLG*, *PROC*, *PROS1*, *RASGRP2*, *RBM8A*, *RUNX1*, *SERPINC1*, *SERPIND1*, *SERPINE1*, *SERPINF2*, *STIM1*, *STXBP2*, *TBXA2R*, *TBXAS1*, *THBD*, *THPO*, *VIPAS39*, *VKORC1*, *VPS33B*, *VWF*, *WAS*

Pulmonary Arterial Hypertension (PAH)
ACVRL1, *BMPR2*, *CAV1*, *EIF2AK4*, *ENG*, *KCNK3*, *SMAD1*, *SMAD4*, *SMAD9*

Primary Immune Disorders (PID)
ADA, *AICDA*, *AIRE*, *AK2*, *AP3B1*, *ATM*, *BLM*, *C1QC*, *C2*, *C4B*, *C5*, *C6*, *C7*, *C8A*, *C8B*, *C8G*, *C9*, *CARD11*, *CARD9*, *CASP10*, *CD19*, *CD27*, *CD3D*, *CD3E*, *CD40*, *CFD*, *CFH*, *CFI*, *CFP*, *CHD7*, *CIITA*, *CORO1A*, *CTLA4*, *CXCR4*, *CYBA*, *CYBB*, *DCLRE1C*, *DKC1*, *DNMT3B*, *DOCK8*, *ELANE*, *F12*, *FAS*, *FERMT3*, *FOXP3*, *G6PC3*, *GATA2*, *HAX1*, *IFNGR1*, *IFNGR2*, *IKBKB*, *IKBKG*, *IL10*, *IL10RA*, *IL10RB*, *IL12B*, *IL12RB1*, *IL2RA*, *IL2RG*, *IL7R*, *IRAK4*, *IRF8*, *ISG15*, *ITK*, *JAGN1*, *JAK3*, *KRAS*, *LCK*, *LIG1*, *LIG4*, *LRBA*, *LYST*, *MAGT1*, *MBL2*, *MEFV*, *MPO*, *MRE11A*, *MVK*, *MYD88*, *NBN*, *NCF1*, *NCF2*, *NCF4*, *NFKB2*, *NFKBIA*, *NHEJ1*, *NHP2*, *NLRP3*, *NOP10*, *ORAI1*, *PGM3*, *PIK3CD*, *PNP*, *PRF1*, *PRKCD*, *PSMB8*, *RAB27A*, *RAG1*, *RAG2*, *RBCK1*, *RFX5*, *RFXANK*, *RFXAP*, *RPSA*, *RTEL1*, *SERPING1*, *SH2D1A*, *SLC29A3*, *SMARCAL1*, *STAT1*, *STAT3*, *STAT5B*, *STIM1*, *STK4*, *STX11*, *STXBP2*, *TAP1*, *TAP2*, *TAPBP*, *TBX1*, *TCN2*, *TERT*, *TINF2*, *TNFRSF1A*, *TTC7A*, *UNC13D*, *VPS45*, *WAS*, *XIAP*, *ZAP70*, *ZBTB24*

Specialist Pathology: Evaluating Exomes in Diagnostics (SPEED) - Neurological
AAAS, *ABAT*, *ABCB7*, *ABCC9*, *ABCD1*, *ABHD5*, *ACAD9*, *ACADM*, *ACADS*, *ACAT1*, *ACOX1*, *ACTB*, *ACY1*, *ADCK3*, *ADSL*, *AFF2*, *AFG3L2*, *AGA*, *AGK*, *AGL*, *AKT1*, *ALDH18A1*, *ALDH3A2*, *ALDH4A1*, *ALDH5A1*, *ALDH7A1*, *ALDOA*, *ALDOB*, *ALMS1*, *ALPL*, *ALS2*, *ALX1*, *ALX3*, *AMER1*, *AMPD2*, *AMT*, *ANKRD11*, *ANO3*, *AP4B1*, *AP4E1*, *AP4M1*, *AP4S1*, *AP5Z1*, *APOPT1*, *APTX*, *ARG1*, *ARID1A*, *ARL6*, *ARSA*, *ARSE*, *ARX*, *ASAH1*, *ASL*, *ASPA*, *ASPM*, *ASXL1*, *ATIC*, *ATL1*, *ATM*, *ATN1*, *ATP13A2*, *ATP1A3*, *ATP7A*, *ATP7B*, *ATRX*, *ATXN2*, *ATXN3*, *AUH*, *B3GALT6*, *B4GALNT1*, *B4GALT7*, *BBS1*, *BBS10*, *BBS12*, *BBS2*, *BBS4*, *BBS5*, *BBS7*, *BBS9*, *BCKDHA*, *BCKDHB*, *BCOR*, *BICD2*, *BIN1*, *BLM*, *BMP4*, *BMPER*, *BRAF*, *BRAT1*, *BRCA2*, *BRIP1*, *BRWD3*, *BSCL2*, *BSND*, *BTD*, *BUB1B*, *C12orf65*, *C19orf12*, *C2orf71*, *C5orf42*, *C9orf72*, *CA2*, *CA8*, *CASK*, *CBS*, *CC2D1A*, *CC2D2A*, *CCBE1*, *CCND2*, *CCT5*, *CDC6*, *CDH15*, *CDKL5*, *CDON*, *CDT1*, *CENPJ*, *CEP290*, *CEP41*, *CEP57*, *CHD7*, *CHRNA4*, *CHST14*, *CHST3*, *CHUK*, *CIB2*, *CKAP2L*, *CLN3*, *CLN5*, *CLN6*, *CLN8*, *CNTNAP2*, *COL11A2*, *COL1A1*, *COL2A1*, *COL4A1*, *COL4A2*, *COLEC11*, *COQ9*, *COX10*, *COX15*, *COX6B1*, *COX7B*, *CPS1*, *CRB1*, *CREBBP*, *CSF1R*, *CSTB*, *CTC1*, *CTDP1*, *CTNS*, *CTSA*, *CTSD*, *CUL4B*, *CYP27A1*, *CYP2U1*, *CYP7B1*, *DAG1*, *DARS2*, *DBT*, *DCTN1*, *DCX*, *DDC*, *DDHD1*, *DDHD2*, *DDOST*, *DDR2*, *DDX11*, *DHCR7*, *DHFR*, *DIS3L2*, *DLAT*, *DLD*, *DMD*, *DMPK*, *DNMT3B*, *DOCK8*, *DOLK*, *DPAGT1*, *DPM1*, *DRD2*, *DYM*, *EBP*, *EFNB1*, *EFTUD2*, *EGR2*, *EHMT1*, *EIF2AK3*, *EIF4G1*, *ELAC2*, *ELOVL4*, *EP300*, *EPG5*, *ERCC2*, *ERCC3*, *ERCC4*, *ERCC6*, *ERCC8*, *ERLIN2*, *ESCO2*, *ETHE1*, *EVC*, *EVC2*, *EXOSC3*, *EXT1*, *EYA1*, *EZH2*, *FA2H*, *FAM111A*, *FAM126A*, *FAM20C*, *FANCA*, *FANCC*, *FANCD2*, *FANCE*, *FBN1*, *FBN2*, *FBP1*, *FBXO7*, *FGD1*, *FGD4*, *FGF3*, *FGFR1*, *FGFR2*, *FGFR3*, *FH*, *FIG4*, *FKRP*, *FKTN*, *FLNA*, *FLNB*, *FLVCR1*, *FMR1*, *FOLR1*, *FOXG1*, *FOXRED1*, *FRAS1*, *FREM2*, *FTCD*, *FTL*, *FTSJ1*, *FUCA1*, *GABRA1*, *GABRB3*, *GABRG2*, *GAD1*, *GALC*, *GALE*, *GALT*, *GAMT*, *GATA6*, *GATM*, *GBA*, *GBA2*, *GCDH*, *GCH1*, *GDAP1*, *GFAP*, *GFM1*, *GJA1*, *GJB1*, *GJC2*, *GK*, *GLB1*, *GLDC*, *GLI3*, *GLUD1*, *GLUL*, *GM2A*, *GNAL*, *GNAS*, *GNPAT*, *GNPTAB*, *GNPTG*, *GNS*, *GPR56*, *GRIA3*, *GRIK2*, *GRIN2A*, *GRN*, *GTF2H5*, *GUSB*, *HADH*, *HAX1*, *HCCS*, *HCFC1*, *HDAC4*, *HDAC8*, *HEXA*, *HEXB*, *HGSNAT*, *HK1*, *HOXA1*, *HPRT1*, *HRAS*, *HSD17B10*, *HSD17B4*, *HSPD1*, *HSPG2*, *HTT*, *HUWE1*, *IDS*, *IDUA*, *IFT140*, *IGF1*, *IGF1R*, *IGF2*, *IKBKG*, *IL1RAPL1*, *INPP5E*, *IQSEC2*, *ISPD*, *ITGA7*, *IVD*, *KANSL1*, *KARS*, *KAT6B*, *KBTBD13*, *KCNC3*, *KCNJ10*, *KCNQ2*, *KCNT1*, *KCTD7*, *KDM5C*, *KDM6A*, *KIAA0196*, *KIAA1279*, *KIF11*, *KIF1A*, *KIF1C*, *KIF5A*, *KIF7*, *KIRREL3*, *KMT2A*, *KMT2D*, *KRAS*, *KRIT1*, *L1CAM*, *L2HGDH*, *LAMA2*, *LAMC3*, *LAMP2*, *LARGE*, *LEPRE1*, *LHX3*, *LITAF*, *LMBRD1*, *LMNA*, *LRP2*, *LRP5*, *LRPPRC*, *LRRK2*, *LYST*, *MAN2B1*, *MANBA*, *MAOA*, *MAP2K1*, *MAP2K2*, *MAPT*, *MASP1*, *MC2R*, *MCCC1*, *MCOLN1*, *MCPH1*, *MECP2*, *MED12*, *MEF2C*, *MEGF10*,

MEGF8, MFSD8, MGAT2, MGP, MID1, MITF, MKKS, MKS1, MLC1, MMAA, MMAB, MMACHC, MMADHC, MNX1, MOCS2, MPLKIP, MPV17, MPZ, MRE11A, MT-ATP6, MT-ND4, MT-TK, MTHFR, MTMR2, MT-PAP, MTR, MTRR, MTTP, MUT, MYCN, MYH3, MYO5A, MYO7A, NAGA, NAGLU, NAGS, NBN, NDE1, NDP, NDRG1, NDUFA1, NDUFS1, NDUFS4, NDUFS7, NDUFS8, NDUFV1, NEFL, NEU1, NF1, NFU1, NHS, NIPA1, NIPBL, NKX2-1, NKX2-5, NPC1, NPC2, NPHP1, NRAS, NSD1, NSDHL, NT5C2, NTRK1, NUBPL, OCRL, OFD1, OPA3, OPHN1, ORC1, ORC4, ORC6, OTC, OTX2, PAFAH1B1, PAH, PAK3, PALB2, PANK2, PARK2, PARK7, PAX2, PAX6, PC, PCBD1, PCCA, PCCB, PCDH19, PCNT, PDCD10, PDE4D, PDGFB, PDGFRB, PDHA1, PDHX, PDSS2, PEPD, PEX1, PEX10, PEX12, PEX13, PEX14, PEX16, PEX19, PEX2, PEX26, PEX3, PEX5, PEX6, PEX7, PGAP1, PGK1, PHF6, PHGDH, PIGA, PIGL, PIGO, PIGV, PIK3CA, PIK3R2, PINK1, PITX3, PLA2G6, PLEC, PLOD1, PLP1, PMM2, PMP22, PNKD, PNKP, PNPLA6, PNPO, PNPT1, POC1A, POLG, POMGNT1, POMGNT2, POMT1, POMT2, PORCN, POU1F1, PPP2R2B, PQBP1, PRKAR1A, PRKRA, PROP1, PRPS1, PRRT2, PRSS12, PRX, PSEN1, PSMB8, PSPH, PTCH1, PTDSS1, PTEN, PTPN11, PTS, PYCR1, QDPR, RAB23, RAB39B, RAB3GAP1, RAB3GAP2, RAD21, RAF1, RAI1, RBM8A, RECQL4, REEP1, REEP2, RET, RNASEH2A, RNASEH2B, RNASEH2C, RNASET2, RNU4ATAC, ROGDI, ROR2, RPGRIP1L, RPS6KA3, RTN2, RYR1, SACS, SALL1, SATB2, SBF2, SC5D, SCN1A, SCN1B, SCN4A, SCN8A, SCO1, SCO2, SDHA, SDHAF1, SETBP1, SF3B4, SGCE, SGSH, SH3TC2, SHH, SHOC2, SIGMAR1, SIL1, SIX3, SKI, SLC12A6, SLC16A2, SLC17A5, SLC19A3, SLC20A2, SLC22A5, SLC25A15, SLC25A20, SLC2A1, SLC2A10, SLC33A1, SLC35C1, SLC46A1, SLC4A4, SLC52A3, SLC5A5, SLC6A1, SLC6A17, SLC6A19, SLC6A3, SLC6A5, SLC6A8, SLC9A6, SLX4, SMARCA2, SMARCA4, SMARCAL1, SMARCB1, SMC1A, SMOC1, SMPD1, SNCA, SOX10, SOX2, SOX3, SPAST, SPG11, SPG20, SPG21, SPG7, SPR, SPRED1, SRD5A3, STRA6, STS, STXBP1, SUMF1, SURF1, SYNGAP1, SYNJ1, SYP, TAF1, TARDBP, TAT, TAZ, TBC1D24, TBCE, TBP, TBX1, TCF4, TCOF1, TECPR2, TFAP2A, TFAP2B, TFG, TGFBR1, TH, THAP1, TIMM8A, TMCO1, TMEM165, TMEM237, TMEM67, TMEM70, TOR1A, TP63, TPP1, TRAPPC9, TREX1, TRIM32, TRIM37, TSC1, TSC2, TSPAN7, TTC19, TTC8, TUBA1A, TUBA8, TUBB2B, TUBB4A, TUSC3, TWIST1, TYR, UBE3A, UBR1, UGT1A1, UMPS, UPF3B, UROC1, VAMP1, VDR, VIPAS39, VLDLR, VPS35, WDPCP, WDR45, WDR62, WNT5A, XPA, ZBTB20, ZC4H2, ZDHHC9, ZEB2, ZFYVE26, ZIC2, ZNF711

Specialist Pathology: Evaluating Exomes in Diagnostics (SPEED) - Retinal Dystrophy
ABCA4, ABCC6, ABHD12, ACBD5, ADAM9, ADAMTS18, AHI1, AIPL1, ALMS1, ARL2BP, ARL6, ARMS2, ATF6, ATXN7, BBIP1, BBS1, BBS10, BBS12, BBS2, BBS4, BBS5, BBS7, BBS9, BEST1, C12orf65, C1QTNF5, C2, C21orf2, C2orf71, C3, C8orf37, CA4, CABP4, CACNA1F, CACNA2D4, CAPN5, CC2D2A, CDH23, CDH3, CDHR1, CEP164, CEP250, CEP290, CERKL, CFB, CFH, CHM, CIB2, CLN3, CLRN1, CNGA1, CNGA3, CNGB1, CNGB3, CNNM4, COL11A1, COL2A1, COL9A1, CRB1, CRX, CSPP1, CYP4V2, DFNB31, DHDDS, DHX38, DMD, DRAM2, DTHD1, EFEMP1, ELOVL4, EMC1, ERCC6, EYS, FAM161A, FBLN5, FLVCR1, FSCN2, FZD4, GDF6, GNAT1, GNAT2, GNPTG, GPR179, GRK1, GRM6, GUCA1A, GUCA1B, GUCY2D, HARS, HGSNAT, HK1, HMCN1, HMX1, HTRA1, IDH3B, IFT140, IFT172, IFT27, IMPDH1, IMPG1, IMPG2, INPP5E, INVS, IQCB1, ITM2B, JAG1, KCNJ13, KCNV2, KIAA1549, KIF11, KIZ, KLHL7, LAMA1, LCA5, LRAT, LRIT3, LRP5, LZTFL1, MAK, MERTK, MFN2, MFRP, MKKS, MKS1, MT-ATP6, MTTP, MVK, MYO7A, NDP, NEK2, NEUROD1, NMNAT1, NPHP1, NPHP3, NPHP4, NR2E3, NR2F1, NRL, NYX, OAT, OFD1, OPA1, OPA3, OPN1LW, OPN1MW, OPN1SW, OR2W3, OTX2, PANK2, PAX2, PCDH15, PCYT1A, PDE6A, PDE6B, PDE6C, PDE6G, PDE6H, PDZD7, PEX1, PEX2, PEX7, PGK1, PHYH, PITPNM3, PLA2G5, PLK4, PNPLA6, POC1B, PRCD, PRDM13, PROM1, PRPF3, PRPF31, PRPF4, PRPF6, PRPF8, PRPH2, PRPS1, RAB28, RAX2, RB1, RBP3, RBP4, RD3, RDH11, RDH12, RDH5, RGR, RGS9, RGS9BP, RHO, RIMS1, RLBP1, ROM1, RP1, RP1L1, RP2, RP9, RPE65, RPGR, RPGRIP1, RPGRIP1L, RS1, SAG, SDCCAG8, SEMA4A, SLC24A1, SLC7A14, SNRNP200, SPATA7, SPP2, TEAD1, TIMM8A, TIMP3, TLR3, TLR4, TMEM126A, TMEM237, TOPORS, TREX1, TRIM32, TRPM1, TSPAN12, TTC8, TTLL5, TTPA, TUB, TUBGCP4, TUBGCP6, TULP1, UNC119, USH1C, USH1G, USH2A, VCAN, WDPCP, WDR19, WFS1, ZNF408, ZNF423, ZNF513
